# A Statistical Study of Serum Cholesterol Level by Gender and Race 

**Published:** 2017-07-25

**Authors:** Bhikhari Prasad Tharu, Chris P. Tsokos

**Affiliations:** ^1^ Department of Mathematics, Spelman College, Atlanta, GA, USA; ^2^ Department of Mathematics and Statistics, University of South Florida, Tampa, FL, USA

**Keywords:** Cholesterol, Malnutrition, heart disease

## Abstract

**Background:** Cholesterol level (CL) is growing concerned as health issue in human health since it is considered one of the causes in heart diseases. A study of cholesterol level can provide insight about its nature and characteristics.

**Study design:** A cross-sectional study.

**Methods:** National Health and Nutrition Examination Survey (NHANS) II was conducted on a probability sample of approximately 28,000 persons in the USA and cholesterol level is obtained from laboratory results. Samples were selected so that certain population groups thought to be at high risk of malnutrition. Study included 11,864 persons for CL cases with 9,602 males and 2,262 females with races: whites, blacks, and others. Non-parametric statistical tests and goodness of fit test have been used to identify probability distributions.

**Results:** The study concludes that the cholesterol level exhibits significant racial and gender differences in terms of probability distributions. The study has concluded that white people are relatively higher at risk than black people to have risk line and high-risk cholesterol. The study clearly indicates that black males normally have higher cholesterol. Females have lower variation in cholesterol than males.

**Conclusions:** There exists gender and racial discrepancies in cholesterol which has been identified as lognormal and gamma probability distributions. White individuals seem to be at a higher risk of having high-risk cholesterol level than blacks. Females tend to have higher variation in cholesterol level than males.

## Introduction

Cholesterol is a growing issue because of its impact on human health^1, 2^. Cigarette smoking, high blood pressure, and high blood cholesterol are the most clearly established risk factors that have been identified as being strongly associated with coronary heart disease (CHD)^2-5^. Total serum cholesterol level (SCL) is a major risk factor for CHD which is the leading cause of death in the United State ^6-8^. CHD is responsible for more deaths than all forms of cancer combined ^3, 5^. A recommendation has been made that total SCL for adults should be below 200mg/dl and individuals with values between 200mg/dl to 239 mg/dl should be considered as borderline high risk; those with values more than 240 mg/dl should be regarded as high risk for CHD ^3, 9^. Hence, a detailed study of SCL is essential for public health.

A better understanding of lipoprotein production and removal, lipoprotein receptors, and apolipoproteins is needed because they are considered the most important factors in cholesterol. Various studies have been carefully executed to reduce SCL through different means through diets and drugs ^10, 11^. Significant positive changes have been achieved through dietary means as well as through drugs in reducing cholesterol level in test subjects. Relevant of effects on SCL can be carefully handled through drugs that have previously been recognized. Studies have been carried out in order to address these issues as well ^12-14^. “*Attempts have been made to make predictions about the SCL based on age in order to better understand the relationship between age and cholesterol level*” ^15-17^.

Precisely defined diets and pharmacologic interventions to reduce blood cholesterol and other lipids are presently being studied in individuals under carefully controlled conditions to investigate effects of drugs on SCL ^18^. The compounds that are more effective, economical, and safe for people in the reduction of blood cholesterol are under intensive research. Longevity of life in the elderly population who have a high-density lipoprotein cholesterol has also been investigated in order to better understand its effects on survival.

That being said, there are very few studies that have been carried out addressing SCL, based on gender and race^19^. SCL is heavily dependent on two factors: it is strongly influenced by food intake of an individual^20, 21^ and it varies by race^21^. In addition, the resistance to disease capabilities varies by gender and race as well^16, 21, 22^. It is therefore equally important for the study of SCL to take both factors into consideration.

In this paper, we performed statistical analysis of the SCL and statistically discuss behavior based on gender and race. We identified the probability distributions that best describe the cholesterol level for different genders and races. Such a characterization will be crucial in obtaining central tendency, dispersion, skewness, and kurtosis of distributions. Primarily, this study attempts to estimate the probability that a randomly selected person`s cholesterol level is at normal, at borderline risk, and at high risk with respect to gender and race which has not been done in this field to the best of our knowledge. Our study will be able to provide further insight through this analysis into its nature and will assist in exploring SCL`s various aspects. Succinctly stated, the purpose of this study is to enable researchers to identify subgroups of the population who are at risk with respect to SCL^19^ and to identify distributional differences among the population subgroups of epidemiological interest. The analysis has been performed using statistical software `R` and the hypothesis has been tested at 10% level of significance.

## Methods

The data utilized in this paper were made available by the inter-university Consortium for Political and Social Research and the data for National Health and Nutrition Examination Survey (NHANS) II, 1976-1980: Serum Cholesterol was originally collected by United States Department of Health and Human Services.

NHANS II was conducted on a nationwide probability sample of approximately 28,000 persons. The target population for the survey was the civilian noninstitutionalized population of the United States (including Alaska and Hawaii). The NHANES II serum cholesterol data files contain two parts of the extensive data available. One part consists of the demographic information obtained from household interview and the other part is laboratory results. The survey started in February 1976 and was completed in February 1980. Samples were selected so that certain population groups thought to be at high risk of malnutrition (persons with low incomes, preschool children, and the elderly) were oversampled. Adjusted sampling weights were then conducted for persons over the age of 76, sex, and race categories in order to inflate the sample in such a manner as to closely reflect the estimated civilian noninstitutionalized U.S. population.

**Figure 1:**
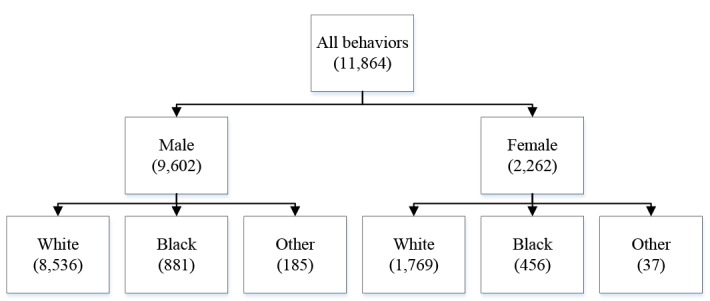
Distribution of individuals under study based on sex and race

In addition to the general examination components, several more detailed examinations were performed on subsamples of the population. Our study included 11,864 persons for SCL cases with 9,602 males and 2,262 females. The information relating to SCL in NHANS II survey considered codes 355-357. Primary site codes were 1 and 2 for males and females respectively. Similarly, codes for white, black, and other were 1, 2, and 3 respectively. Male data included 8,536 white, 881 black, and 185 other individuals. On the other hand, Female data included 1,769 white, 456 black, and 37 other individuals. Descriptive information for the total number of individuals by sex and race is presented in schematic diagram (Figure 1) which explains how the study was systematically organized.

In order to perform parametric analysis of cholesterol levels, the probability distributions that best fit the cholesterol data are lognormal (a,c) and gamma distribution (b). The probability distributions were confirmed, taking into consideration all available data. Kolmogorov Smirnov goodness of fit test was performed to identify the underlying distributions. With respect to the lognormal distribution (a) characterizes the cholesterol levels of most of the male cases. Gamma probability distribution represents cholesterol levels of mostly female cases, which is shown in equation (b). Similarly, the probability density function of three-parameter lognormal distribution is given by equation (c).

(a) $$\text{f(x)=\{}\frac{1}{\sqrt{2\pi \sigma x}}\exp\underset{\underset{}{0,}}{\left(-\frac{{\left[\ln(x)-\mu \right]}^{2}}{2\sigma^{2}}\right)},\underset{\underset{}{Otherwise}}{x,\sigma \in (0,\infty )},-\infty \leq \mu \leq \infty$$

where μ is location parameter, σ shape parameter, and x is random variable.

(b) $$\text{g(x)=}\{\underset{\underset{}{0,}}{\frac{1}{\beta \gamma \Gamma \gamma }}X^{\gamma -1}\exp(-\frac{x}{B}),\;\underset{\underset{}{Otherwise}}{x,\gamma ,\beta }>0$$

where γ is shape parameter, β is scale parameter, and x is random variable.

Where $\Gamma \gamma =\int_{0}^{\infty }x^{\gamma -1}e^{-x}dx,\;\gamma \in (0,\infty )$

(c) $$\text{h(x)=}\{\underset{\underset{}{0}}{\frac{1}{(x-y)\sigma \sqrt{2\pi }}}\exp\underset{\underset{}{,}}{\frac{{\left[\ln(x-y)-\mu \right]}^{2}}{2\sigma^{2}}},\gamma <x<\infty >0,\underset{\underset{}{Otherwise}}{-\infty \leq \mu \leq \infty }$$

where μ is location parameter, σ shape parameter, γ is scale parameter, and x is random variable.

## Results

In this section, cholesterol level by sex and race was investigated. Distinctively different cholesterol levels may have important implications. All pairwise comparisons were performed non-parametrically, not relying on any particular distribution (Table 1). The mean cholesterol levels of males and females for overall behaviors were investigated. The study performed the Mann-Whitney-Wilcoxon nonparametric test in order to identify whether or not the males and females were from two identical population distributions, with respect to cholesterol level (Table 1).

**Table 1:** The significant test of comparisons with *P* values

**Variables**	**Tests**	***P*** ** value**
Male vs female	Mann-Whitney-Wilcoxon	0.001
Male		
All races	Kruskal-Wallis	0.013
Black vs white	Mann-Whitney-Wilcoxon	0.007
Black vs other	Mann-Whitney-Wilcoxon	0.009
White vs other	Mann-Whitney-Wilcoxon	0.089
Female		
All races	Kruskal-Wallis	0.006
Black vs white	Mann-Whitney-Wilcoxon	0.001
Black vs other	Mann-Whitney-Wilcoxon	0.061
White vs other	Mann-Whitney-Wilcoxon	0.065

The authors were able to reject the null hypothesis that male and female cholesterol levels were coming from two identical populations (*P*=4.8E-09). It was therefore, crucial to investigate male and female, independently. The authors also examined the equality of mean cholesterol levels for white, black, and other in each of the cases for males and females, to understand if they were coming from identical populations. All comparisons were made using the non-parametric Kruskal-Wallis test and Mann-Whitney-Wilcoxon test. In each case, we were able to reject the null hypothesis (Table 1) with a *P*=0.013 for males and a *P*=0.006 for females and concluded that the cholesterol levels were coming from independent populations with different means. The authors also tested, pairwise, a non- parametric test for white, black, and other. The null hypothesis was rejected in each of those cases. Nonparametric test with corresponding p-values have been reported (Table 1).

Once the study identified racially classified populations from both males and females that exhibit different subpopulations, the study performed the Kolmogorov goodness of fit test to determine the underlying distribution the data to follow. The study identified the overall male cholesterol level that exhibited lognormal probability distribution (a) whereas the overall female population exhibited gamma probability distribution (b). Similarly, the study identified and concluded that white males followed three parameters lognormal probability distribution (c), black males followed lognormal probability distribution (a), and males other resembled gamma probability distribution (b). In addition, the study determined that white females exhibited gamma distribution (b), black females exhibited lognormal distribution (a), and that the female others satisfied the Burr distribution. Since the sample size for Burr distribution was very small, the study makes a disclaimer for careful interpretation. Estimated parameters for fitted distributions are presented (Table 2).

**Table 2. The identified probability distributions with P value and the estimated parameters:**
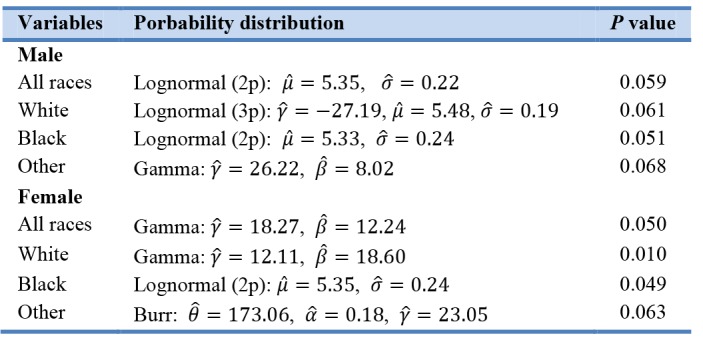


**Figure 2:**
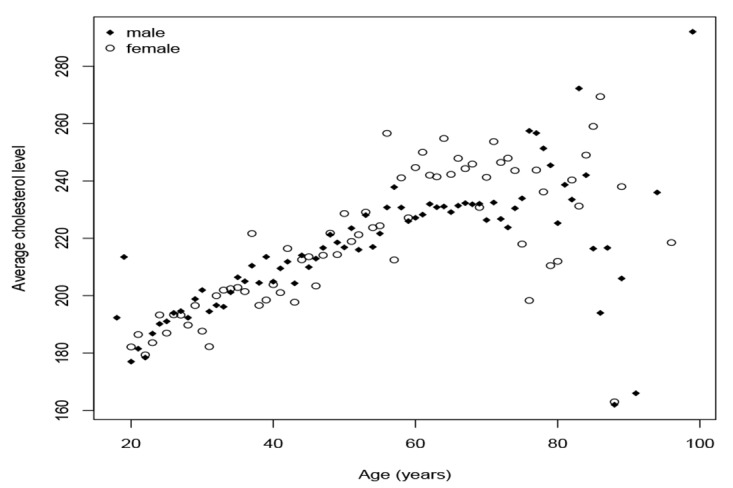
Age specific average cholesterol levels by gender

Age specific average cholesterol levels by sex were plotted to present the variability for various ages. Cholesterol levels varied greatly in both age and sex. Mean cholesterol levels were computed and compared statistically by gender specific averages (Figure 2). Clearly, the average cholesterol level of an individual over 60 years of age exhibited greater variability than those under the age of 60 and the average was consistent in both males and females. Mean cholesterol levels were progressively and consistently higher in each succeeding age, prior to the age of 60. This was true for both males and females. The SCL appeared to be less volatile for ages under 60 years old and displayed a clear linearly increasing pattern for both genders. There was no clear pattern of cholesterol levels after the age of 60 for both genders. However, cholesterol level for females appeared to be consistently higher than males. The study noted that above 80 years old there were relatively few data values, as compared to other ages.

**Figure 3:**
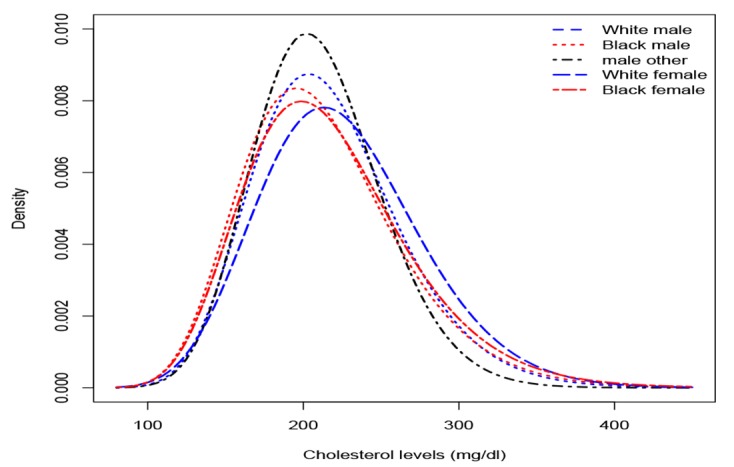
Plots of identified probability density functions (pdf) of cholesterol level

The study plotted probability density functions (Figure 3) that best described the cholesterol level for races, for both males and females. There was a clear difference between cholesterol levels of males and females. The distributions that SCL exhibited were skewed to the right with different means and different skewness. Having known the underlying distributions, we might have a better understanding of the variability of cholesterol level and estimates of basic statistics from which we would have been able to draw proper inferences for different subpopulations.

Statistics based on distributions have been presented (Table 3). Such measurements and analysis are common but have great implications in evaluations and treatments. Females appear to have a higher cholesterol level, on average, than males and the higher cholesterol level is consistent in all the races. This finding is also applicable for variance. Significantly, however, white individuals, both males and females, appear to have higher cholesterol levels than other subpopulations. White females have the highest average cholesterol level among all and have the highest kurtosis. Higher kurtosis implies more of a variance due to infrequent extreme deviation from the mean. It was determined that white females have the highest extreme variation from the mean cholesterol level.

**Table 3:** Estimated central tendency and variability of cholesterol level classified as race and gender using estimated parameters of fitted distributions

**Variables**	**Mean**		**Variance**		**Skewness**		**Kurtosis**	
	**Male**	**Female**	**Male**	**Female**	**Male**	**Female**	**Male**	**Female**
All races	216.26	223.60	2345.47	2736.34	0.65	1.24	4.25	11.46
White	216.71	232.25	2285.80	2728.39	0.68	-86.77	4.49	384.16
Black	212.83	217.29	2629.64	2897.67	7.20	-63.97	34.75	255.86
Other	210.26	223.80	1769.04	2567.45	0.11	0.80	2.28	2.61

We have observed (Table 4) that black males are 5% more likely to have normal cholesterol level than white males whereas it is nearly 7% in females. Table 4 shows that the probability of risk line cholesterol for white is higher than black and is consistent in males and females. It is also true in high risk level of cholesterol level. Females appear to develop high risk level cholesterol level more than males and it seems to be true for all races. White females have significantly high risk of cholesterol level.

**Table 4:** Estimated probabilities of normal, line of risk, and high risk of total cholesterol level classified at race and gender

**Variables**	**Normal (**≤**199)**		**Risk line (200-239)**		**High risk (**≥**240)**	
	**Male**	**Female**	**Male**	**Female**	**Male**	**Female**
Black	0.4348	0.4059	0.2849	0.2819	0.2662	0.2983
White	0.3855	0.3280	0.3138	0.2956	0.2854	0.3619
Others	0.4152	0.4437	0.3436	0.2832	0.2246	0.2575

## Discussion

The study identified the probability distributions for males and females that are respectively lognormal and gamma, which were included in the study. A probability model was applied to investigate its effect on SCL. The study will help researchers to have a greater insight into risk factors and their behaviors. Majority of the individuals in the data set were white and had a greater variability in both males and females. The differences in distributions might be used as benchmarks for racial and gender comparisons, and as possible indicators of changes in factors known to influence serum cholesterol, such as diet and drugs.

The authors witnessed that SCL increased as age increased and it is possible that SCL becomes more volatile as an individual becomes older most notably when above 60 years old (Figure 2). “*SCL is most likely to develop during the late teens to mid-40s*”^23, 24^. Different behaviors between the race-sex groups were observed in our study. The authors identified a resemblance to different probability distributions. The authors` findings are supported by research ^23^. The higher the level of cholesterol that an individual has, the greater the risk of subsequently developing CHD would be. The findings of the study were proven with prospective studies such as the Framingham Study ^25^. Since the likelihood of developing high SCL is dependent upon race and gender, borderline high risk and high risk level of SCL are likely to be different^19^, according to race and gender. This observation may be subject to a further area of research. The deeper one delves into the issues, the more useful information will be obtained. Further information derived from future research will help researchers speculate more precisely about SCL^19^.

Our study suggests that the average SCL for males and females is different indicating that to a greater extent, reliance upon the analytical precision and accuracy of laboratory measurements will be required before making a generalized assumption about the degree of risk of an individual. An informed decision which is based on male information cannot be generalized to the female and vise-versa ^23^. It has been clinically proven that lowering elevated cholesterol levels will reduce the risk of CHD. The degree of risk is relative to gender and race. Efforts have been made to investigate the relationship of some demographic variables to SCL as they may relate to SCL. Our study will assist in developing guidelines, which will better inform physicians and public health practitioners of best practices, when determining how to best treat an individual, dependent upon gender and race. The results of this study will inform individuals and be an aid in preventing premature deaths^7^. Further, the study provides data, which will aid in better understanding the relationship of certain risk factors to the development of high SCL. The study pivots on aspects of gender and race.

The study identified differences in probability distributions with respect to gender and race to characterize SCL, which in turn was dependent upon food intake. Therefore, more careful studies of diet, eating patterns, as well as the attitudes and life style of general population and specific subpopulations will help to better understand their relative relationship to SCL. “*A number of retrospective case-control and cohort studies have investigated the associations between intakes of dietary fats and cholesterol and their associated risks*” ^26, 27^. Since these habits can vary the results, based upon on race and gender, inferences made, which do not take into account these factors, will be misleading which will most likely have serious consequences^28^.

We have observed that the probability of having higher cholesterol level for black people is relatively more than other and white people (Table 4). However, the probability of having risk level of cholesterol and high risk level of cholesterol is more in white people than black. It has been found that blacks have a greater incidence of hypertension. However, the rate of coronary heart disease among black is not higher than white^29-31^. It has been found in various researches that plasma triglyceride levels are consistently lower in Blacks than in Whites^32-35^. “*HDL cholesterol is approximately 20% higher in African American men than in white men*” ^31-34, 36-38^. It has been also found that socioeconomic status is not associated with coronary heart disease^39^ which is a measure factor in many blacks^40^. More detailed research could provide light how black people have few incidence of coronary heart disease than white despite relatively higher level of cholesterol level.

## Conclusions

Lognormal and gamma probability distributions with different parameter estimates represent differences in cholesterol level regarding gender and race. Moreover, there exists high variation in cholesterol level among females than males. High risk level of cholesterol appears to be more in whites than blacks. In addition, females likely to have high risk level of cholesterol than males which is consistent throughout races.

## Acknowledgment

The authors are thankful to the data provider. The data utilized in this study were made available by the Inter- University Consortium for Political and Social Research. The data for National Health and Nutrition Examination Survey II, 1976-1980: Serum Cholesterol was originally collected by United States Department of Health and Human Services: National Center for Health Statistics. The authors would like to express their appreciation to the associate editor and two anonymous reviewers for their valuable comments.

## Conflict of interest statement

We wish to confirm that there are no known conflicts of interest associated with this publication.

## Funding

There has been no financial support for this work that could have influenced its outcome.

## Highlights

- High risk of cholesterol level appears to be higher in whites than blacks.
- Variation in cholesterol level in females is higher than males.
- Females are more at risk for high cholesterol than males, which is consistent in races.
